# Composition of Whole Grain Dietary Fiber and Phenolics and Their Impact on Markers of Inflammation

**DOI:** 10.3390/nu16071047

**Published:** 2024-04-03

**Authors:** Jabir Khan, Palwasha Gul, Muhammad Tayyab Rashid, Qingyun Li, Kunlun Liu

**Affiliations:** 1 College of Food Science and Engineering, Henan University of Technology, Zhengzhou 450001, China; jabirkhan@163.com (J.K.); palwasha.gull68@outlook.com (P.G.); trashid208@gmail.com (M.T.R.); qyli2021@163.com (Q.L.); 2 School of Food and Strategic Reserves, Henan University of Technology, Zhengzhou 450001, China

**Keywords:** whole grains, dietary fiber, phenolic acids, health benefits, anti-inflammation

## Abstract

Inflammation is an important biological response to any tissue injury. The immune system responds to any stimulus, such as irritation, damage, or infection, by releasing pro-inflammatory cytokines. The overproduction of pro-inflammatory cytokines can lead to several diseases, e.g., cardiovascular diseases, joint disorders, cancer, and allergies. Emerging science suggests that whole grains may lower the markers of inflammation. Whole grains are a significant source of dietary fiber and phenolic acids, which have an inverse association with the risk of inflammation. Both cereals and pseudo-cereals are rich in dietary fiber, e.g., arabinoxylan and β-glucan, and phenolic acids, e.g., hydroxycinnamic acids and hydroxybenzoic acids, which are predominantly present in the bran layer. However, the biological mechanisms underlying the widely reported association between whole grain consumption and a lower risk of disease are not fully understood. The modulatory effects of whole grains on inflammation are likely to be influenced by several mechanisms including the effect of dietary fiber and phenolic acids. While some of these effects are direct, others involve the gut microbiota, which transforms important bioactive substances into more beneficial metabolites that modulate the inflammatory signaling pathways. Therefore, the purpose of this review is twofold: first, it discusses whole grain dietary fiber and phenolic acids and highlights their potential; second, it examines the health benefits of these components and their impacts on subclinical inflammation markers, including the role of the gut microbiota. Overall, while there is promising evidence for the anti-inflammatory properties of whole grains, further research is needed to understand their effects fully.

## 1. Introduction

According to the American Association of Cereal Chemists International (AACCI) 1999 [1], “whole grains include intact, crushed, cracked, and flaked kernels containing starchy endosperm, germ, and bran in the same proportions as the intact kernel”. However, in 2006, pseudo-cereals were included in the category of whole grains [2] due to the fact that they are used in the same traditional way as cereals and have a macronutrient composition that is largely similar to that of cereals. Whole grain intake, rather than that of refined grains, is known to have numerous health benefits, many of which are attributed solely to the presence of dietary fiber [3]: first, by releasing indigestible fiber, which affects the composition and activity of the gut microbiota; second, by providing substrates like resistant starch and non-starch polysaccharides. However, other components, like phenolic acids, are also likely involved given that they can be metabolized into useful metabolites of microbiota [4]. Previous observational studies have found an inverse association between the intake of whole grains and markers of inflammation [5,6,7]; however, the data from existing studies are inconsistent [8,9]. Additionally, different studies have been conducted to investigate the association between refined grain consumption and inflammatory markers. However, the evidence is inconsistent regarding the consumption of refined grains and health outcomes; the majority of research has shown that consuming refined grains has either negative or neutral effects on inflammation and disease outcomes [10,11,12]. Furthermore, refined grains have also been linked to unhealthy eating habits that increase the likelihood of developing certain diseases [13,14].

To date, several epidemiological studies have been conducted on the association between whole grain intake and several chronic diseases, such as inflammation [4,5,6,7,15]. According to Browning et al. [16], their study on whole grains and inflammation makers found that inflammatory markers like C-reactive protein (CRP), interleukin-6 (IL-6), and tumor necrosis factors (TNF) can be used to assess the anti-inflammatory properties of whole grains and might be downregulated as an inflammatory response [17]. According to this study, the association between inflammation and illness may be larger than previously thought since some inflammatory indicators, like CRP, have significant within-individual variability. Most observational and interventional studies have focused on the inflammatory biomarkers CRP, TNF-α, TNF-α receptor 1 and 2, IL-6, fibrinogen, and IL-1β. These biomarkers are known to be associated with inflammation, which can lead to the development of neurodegenerative diseases, type 2 diabetes (T2D), joint disorders, cardiovascular disease (CVD), cancer, and allergies [18,19,20]. In particular, IL-6, an acute-phase protein produced by the liver in response to IL-6, appears to be the most studied inflammatory biomarker in studies investigating the influence of whole grains on inflammation [21,22]. This recognition has led researchers to investigate the association between whole grain intake and inflammatory process, aiming to reduce the risk of several chronic diseases [17]. On the other hand, many anti-inflammatory drugs that are commonly used might have side effects [23]. For these reasons, slowing down the inflammatory process becomes critical. Whole grain dietary fiber and phenolic acids have attracted scientific attention as they play a significant role in the reduction of the risk of inflammation with no side effects. While there may never be a single path towards the prevention of inflammation, it is possible that the long-term consumption of whole grains as part of a generally healthy dietary pattern may significantly reduce the development of inflammation and associated diseases. As a result, the purpose of this review is twofold: first, it discusses whole grain dietary fiber and phenolic acids and highlights their potential in lowering the risk of inflammation and other diseases; second, it examines the health benefits of the associations between whole grain intake and biomarkers of inflammation, along with the underlying mechanisms. In summary, this paper explores how whole grains may help to reduce inflammatory markers, as the scientific evidence about the anti-inflammatory properties of whole grains is encouraging. Recent evidence suggests that dietary fiber together with phenolic acids may be more beneficial for health than individual components. However, while there is promising evidence for the anti-inflammatory properties of whole grains, further studies are needed to understand their effects fully.

## 2. Materials and Methods

In the current study, we explored and summarized the literature on dietary fiber and phenolic acids in targeted whole grains (Table 1). Then, we examined the literature that was recently published on the intake of whole grains and the development of inflammation. Human studies, observational studies, and intervention studies were searched on PubMed and Google Scholar. The major keywords for the literature were whole grains, dietary fiber, arabinoxylan, β-glucan, phenolic compounds, hydroxycinnamic acids, hydroxybenzoic acids, gut microbiota, and inflammation. Relevant data that have been published in English in reputable peer-reviewed publications were included for discussion. Finally, all materials accessible in the form of books and conference abstracts, and unpublished materials, were excluded.

## 3. Whole Grain Dietary Fiber and Phenolic Acids and Their Health Potential

### 3.1. Dietary Fiber

According to the definition of dietary fiber given by Health Canada, “dietary fiber is a form of carbohydrate, which are classified according to their degree of polymerization, naturally occurring in all plants that are not digested and absorbed by the small intestine” [24]. Dietary fiber can be further classified based on its water solubility, with the two types being soluble dietary fiber (SDF) and insoluble dietary fiber (IDF) [25]. IDF is present in plants as a structural cell wall component made up of cellulose, insoluble hemicelluloses, and lignin, while SDF is made up of a range of non-cellulosic polysaccharides and oligosaccharides [26]. Hemicellulose, a non-cellulosic component of cell walls, made up of heterogenic polysaccharides, is a common form of DF found in grains [27]. Hemicellulose molecules are broadly categorized into four types: xylans, xyloglucans, glucomannans, and mixed linkage β-glucans [28]. Hemicelluloses can be soluble or insoluble depending on their size and structural characteristics (e.g., side chain substitutions and intermolecular crosslinks) [29]. About 70% of the total dietary fiber composition is made up of arabinoxylan (AX) and 20% is made up of mixed linked β-glucan. Consequently, AX is made up of four structural components: non-substituted, mono- and di- substituted Xyl, and O-2 or O-3 [30]. The O-5 position of arabinose residues can be used to esterify ferulic acid. These ferulic acid structures can form links between AX chains, increasing the molecular weight of the compound while lowering its water extractability.

As shown in Table 2, rye has higher dietary fiber content as compared to corn, sorghum, millet, and triticale, ranging from 14 to 21% dry matter [31,32,33,34,35] (Table 2). Rye includes four types of dietary fiber: AX, cellulose, fructan, and β-glucan. The endosperm cell walls contain AX, which accounts for 45% of the total fiber content (i.e., 45% of total dietary fibre content) [36]. Although both rye and quinoa contain AX, the amount and solubility of AX in rye is greater than that in millet [27].

Among cereal grains, rye contains the largest amount of fructans. Fructans are a form of soluble dietary fiber made up of β-D-fructofuranosyl units that may or may not have a terminal glucose residue [34]. Rye fructans can be either linear or branching in structure. The degree of fructan polymerization in rye often varies from 2 to 60 [37]. The amount of dietary fiber in rye varies according to its location within the kernel. The inner endosperm has less dietary fiber (12%), whereas the outer endosperm and bran part contain between 22 and 38% dietary fiber, respectively [38]. The higher levels of dietary fiber found in rye’s outer kernel layers demonstrate the benefits of consuming whole grains. Corn’s dietary fiber composition ranges between 3.7 and 19.9% on a dry matter basis, with IDF accounting for the largest fraction, namely 3 to 14 g/100 g (Table 2) [32,35,39,40,41]. In corn bran, cellulose and hemicellulose make up the majority of the IDF components [41,42]. The TDF of sorghum ranges from 1.5 to 12% [43,44,45], millet has content of 13–14% [46,47,48], and triticale has content of 14–15% on a dry matter basis in terms of g/100 g [38,49,50,51,52].

**Table 2:** Content of total, soluble, and insoluble dietary fiber in targeted grains, g/100 g.

Whole Grain	TDF	IDF	SDF	References
Rye (*Secalecereale* L.)	15.2–20.9	11.1–15.9	3.7–4.5	[31,32]
	14.7–20.9	10.8–15.9	3.4–4.6	[33,34,35]
Corn (*Zea mays* L.)	3.7–8.6	3.1–6.1	0.5–2.5	[39,40]
	13.1–19.6	11.6–14.0	1.5–3.6	[32,35,41]
Sorghum (*Sorghum bicolor*)	7.55–12.3	6.52–7.90	1.05–1.23	[43,44,45]
Millets (*Eleusine coracana* (L.) Gaertn.)	13.0–13.8	12.5–13.5	0.52–0.59	[46,47,48]
Triticale (*Triticosecale* Wittmack)	14.5	6–8	-	[38,49,50]
	14.6	12.0	0.2–1.3	[51,52]
Quinoa (*Chenopodium quinoa* Willd.)	7.0–26.5	-	-	[53,54]
	11.6–21.6	9.9–12.2	0.4–2.9	[55,56,57]

Quinoa is a pseudo-cereal that has a long history of use as a food component and has some very interesting nutritional properties. In the past 10 years, pseudo-cereals have become increasingly popular as ingredients in gluten-free goods [58]. The dietary fiber content of quinoa varies from 7 to 27%, with more than 30% being soluble dietary fiber [53,54,55,56,57] (Table 2). However, Alonso-Miravalles et al. reported that quinoa contains 11–19% of TDF [57]. The TDF content in this study was consistent with that found in other studies of quinoa, e.g., 10.4% [54], 11.7% [59], and 12.7% [60]. Compared to these studies, Alvarez-Jubete et al. [58] reported slightly higher values for TDF, namely 14.2%. Additionally, Nascimento et al. [59] revealed that pseudo-cereals contain seven times more fiber than other grains, like rice. Furthermore, the majority of the dietary fiber in these pseudo-cereals, as determined by a monosaccharide analysis of dietary fiber taken from samples of quinoa and amaranth, is made up of galacturonic acid, arabinose, xylose, glucose, and galactose. The main components of quinoa are soluble and insoluble dietary fiber, and it has been categorized as pectic polysaccharide [61] based on the monosaccharide composition and linkage analyses. Xyloglucans are the second most abundant form of dietary fiber contained in quinoa whole grains.

In summary, whole grain cereals and pseudo-cereals contain a wide variety of dietary fiber types. The dietary fiber and phenolic acid composition of whole grains is very varied across different grains. As seen in Table 2, among the above-studied whole grains, quinoa contains the highest content of total dietary fiber. Rye is particularly rich in AX, while quinoa and millet are known for their health benefits, associated with their main dietary fiber, i.e., β-glucan. One of the primary impacts of soluble dietary fiber is an increase in intestinal viscosity. Insoluble dietary fiber, on the other hand, absorbs more water and helps with feces bulking. It has been found that quinoa has the highest levels of dietary fiber, ranging from 7 to 27%, while sorghum has the lowest levels of dietary fiber among cereals, ranging from 7 to 13%. The rankings are variable in other cereals. Quinoa, rye, corn, triticale, millet, and sorghum have the most TDF, in descending order, as shown in Table 2. In a few studies, both SDF and IDF in triticale and quinoa were undetected. The intake of whole grain dietary fiber can reduce the risk of chronic non-communicable diseases [62,63,64,65,66,67]. Diets rich in whole grains play a significant role in lowering the risk of inflammation [6,7,8,9,19,35]. Additional experimental trials are required to verify the composition of TDF, IDF, and SDF in various whole grains.

### 3.2. Health Potential

The bran is a grain’s nutrient storehouse. The whole grain bran’s chemical composition is very complex. The regular consumption of whole grain dietary fiber may help to lower the risk of numerous diseases [64]. In addition to dietary fiber, the bran also contains a number of nutrients, such as protein, vitamins, minerals, and fats, which have been demonstrated to have a wide range of biological activity and other health benefits in populations that consume cereal-grain-based diets [66,68]. However, choosing a good source becomes difficult due to their wide range of physicochemical properties. One of the major components found in the whole grain bran is dietary fiber. The benefits of dietary fiber for human health have been supported by extensive research conducted over the last three decades [65,67]. The main role of β-glucan in the diet is to decrease blood lipids, specifically serum total and LDL cholesterol, and both of these effects have long-term health advantages [69] (Table 3). According to the authors, it was found that the average cholesterol reduction was 4.4%. Notably, the test samples for the meta-analysis included intact whole oats and oat bran, and the data were obtained from 23 trials that used less than 10 g of dietary fiber daily. Furthermore, whole grain β-glucan has several health benefits. [70], including lowering blood cholesterol and glucose levels, decreasing the glycemic index, prebiotic effects, and improving satiety, all of which aid in the long-term management of heart disease and other chronic non-communicable diseases, as shown in Table 3 [71,72,73,74,75,76,77,78,79,80,81,82,83].

The consumption of whole grain AX has been found to enhance lipid metabolism by lowering the LDL cholesterol levels in the blood, improve colon health by lowering the cancer risk, and improve glycemic management by lowering blood glucose levels [72,73]. AX has been shown in studies to inhibit small intestinal transit, limit starch availability to digestive enzymes, and slow the rate of lumen-to-cell glucose diffusion. Any of these factors might contribute to decreased glucose absorption, hence reducing the postprandial glycemic response [74]. The European Food Safety Authority has verified the health claim that AX intake decreases the rise in glucose after a meal [75]. The significant intake of dietary fiber helps individuals to lose weight and enhances blood glucose levels, immune function, and serum cholesterol levels. It also reduces the likelihood of developing several chronic diseases, including CVD and T2D, as well as certain cancers [67,80,81,82]. Recent research on the health effects of whole grains, particularly their bioactive components, has highlighted their potential as a functional food that can lower the risk of multiple chronic diseases [81]. 

Table 3 summarizes the functional compounds together with their locations in the grain fractions. The findings of several studies show the relationship between the health benefits of cereal-based foods, such as arabinoxylan, β-glucan, and other types of dietary fiber [71,72,73,74,75,76,77,78,79,80,81,82,83]. Numerous studies have been conducted throughout the years to determine the health advantages of the dietary fiber found in cereals and pseudo-cereals. While the specific effects of many other dietary fiber types remain under investigation, the intake of some forms, including β-glucan, has been recommended due to their documented health advantages. It is challenging to find conclusive proof of the health benefits of dietary fiber in lowering the risk of chronic diseases, since these advantages are connected to several factors. Additionally, the amount of dietary fiber in grains varies greatly. It is yet unclear, though, to what extent the dietary fiber included in whole grains contributes to these health advantages. Thus, additional study of the compositions of different types of whole grains, as well as their health advantages, is required to translate the science behind these positive impacts into useful information. 

#### 3.2.1. Phenolic Acids

Phenolic compounds are identified by the presence of one or more aromatic rings connected by one or more hydroxyl groups. Phenolic acids include benzoic and cinnamic acid derivatives. In general, “phenolic acids” are phenols having a single carboxylic acid. However, when defining plant metabolites, it refers to a distinct class of organic acids. These naturally occurring phenolic acids have two different carbon frameworks: hydroxycinnamic and hydroxybenzoic. Although the underlying structure remains the same, the amounts and positions of the hydroxyl groups on the aromatic ring determine the variety. 

In plants, phenolic acids are formed by shikimic acid via the phenylpropanoid pathway, as byproducts of the monolignol pathway, and as the breakdown products of cell wall polymers and lignin in vascular plants [84]. Grains contain three different forms of phenolic acids, conjugated, free, and bound, with the binding form predominating [85,86]. They are typically found in the bran and the embryo cell walls of cereal kernels [69,71]. Hydroxycinnamic acids are aromatic carboxylic acids with a C6–C3 structure. Ferulic, *p*-coumaric, caffeic, and sinapic acids are among the most common hydroxycinnamic acids found in grains (Table 4). Hydroxybenzoic acids have a C6–C1 structure, and *p*-hydroxybenzoic, gallic, vanillic, and syringic acids are abundant in grains. It has been demonstrated that hydroxycinnamic acids are more prevalent in plants than hydroxybenzoic acids [87,88]. Whole grains include phenolic acids such as ferulic, vanillic, caffeic, syringic, and *p*-coumaric acids [68,88,89]. Hydroxycinnamic acids are generated in a variety of plant foods, including coffee beans, tea, maté, berries, citrus, grapes, spinach, beetroots, artichokes, potatoes, tomatoes, and cereals [90]. Cinnamic-related compounds have been shown to have anticancer, anti-tuberculosis, antimalarial, antifungal, antibacterial, antiatherogenic, and antioxidant properties. There are several cinnamic acid isoforms found in nature, with trans-CA (trans-3-phenyl-2-propenoic acid; t-CA) being the most common. Because of its low toxicity, t-CA has been widely employed as an antibacterial/antifungal component in medicine. Furthermore, t-CA is present in triticale, barley, oat, rye, rice, and maize, sorghum, and millet, with millet and quinoa having undetectable levels [91,92,93]. The following literature will cover the composition of phenolic acids, including derivatives of benzoic and cinnamic acids in whole grains, as they are present in the most widely eaten grains. Furthermore, the significance of these phytochemicals to the health advantages of whole grain consumptions is studied.

In Table 5, we present eight phenolic acids in targeted cereal grains, demonstrating that ferulic acid is the most abundant phenolic acid in cereals. It is the product of phenylalanine and tyrosine metabolism and is found primarily in the cell walls of rye, triticale [91], corn [94], sorghum [95], millet [96,97,98], and quinoa [99,100]. According to Table 5 the average ferulic acid (4-hydroxy-3-methoxycinnamic acid; FA) content of these grains ranges from 46.2 to 827.2 g/g dry weight, with rye and millet having the highest levels and triticale having the lowest. *p*-Coumaric acid (3-(4-hydroxyphenyl)-2-propenoic acid; p-CA) has been found in rye [91], corn and triticale [101], millet [97], sorghum [95], and quinoa [100]. The range of average content of *p*-coumaric acid in grains ranges from 43.6 g/g dry weight in sorghum to 340.5 µg/g in corn. Caffeic acid(3,4-dihydroxycinnamic acid) is typically found in foods as an ester with quinic acid to create chlorogenic acid. Caffeic acid can be found in rye, corn and triticale [91], millet and sorghum [95], and quinoa [100]. Sorghum has an average value of 4.6 µg/g dry weight; while sorghum has content of 32.1 µg/g. Sinapic acid (4-hydroxy-3, 5-dimethoxy cinnamic acid; SA) is prevalent in several plants, including rye [91,102], corn and triticale [91,101,103], sorghum [95], and millet [96]. The average content of sinapic acid in cereal grains varies from 8.22 to 94.2 µg/g sorghum and millet, whereas it is undetected in quinoa.

Hydroxybenzoic acids are phytochemicals found in various diets. It should be noted that the circulating hydroxybenzoic acids in humans can be the absorbed products of bacterially mediated polyphenol metabolism in the lower intestine [105,106]. This section discusses the widely discovered hydroxybenzoic acids in grains, namely *p*-hydroxybenzoic acid, gallic acid, vanillic acid, and syringic acid. *p*-Hydroxybenzoic acid is present in rye, corn and triticale [91], sorghum and millet [101], and quinoa [100]. The average amount of *p*-hydroxybenzoic acid ranges 3.0 µg/g in millet to 36.2 µg/g in sorghum. Gallic acid is found in rye, corn and triticale [91], sorghum [95], millet [96,97], and quinoa [100]. Its presence has not been reported in quinoa, and the greatest amount among grains was found in triticale, at 333.7 µg/g. Vanillic acid has been detected in rye, corn, and triticale [91], sorghum [105], millet [103], and quinoa [100]. The average concentration varies from 10.3 µg/g in corn to 446.0 µg/g in triticale. Syringic acid has been found in rye [91], corn and triticale [101], sorghum [95], and millet [104]. The average content of syringic acid is highest in triticale 173.2 µg/g and lowest in rye 6.3 µg/g, whereas it is undetected in quinoa.

In summary, regarding hydroxycinnamic acids and hydroxybenzoic acids (Table 5), ferulic acid is the most abundant in all grains except corn and triticale. In corn, ferulic acid is ranked second and *p*-coumaric acid ranks highest; however, in triticale, ferulic acid is ranked fifth, with vanillic acid, gallic acid, syringic acid, and *p*-coumaric acid being the most prevalent in triticale, respectively. In buckwheat, vanillic acid and gallic acid are predominant, in descending order. The ranking becomes variable for the rest of the phenolic acids within grains. The greatest total content of hydroxycinnamic acids and hydroxybenzoic acids, in descending order, is found in triticale, rye, corn, millet, sorghum, and quinoa; for the four hydroxycinnamic acids, the order is rye, corn, millet, triticale, sorghum, and quinoa; and in the four hydroxybenzoic acids, it is triticale, corn, millet, sorghum, quinoa, and rye. Remarkably, all grains except triticale have a ratio of hydroxycinnamic acids to hydroxybenzoic acids greater than 1. These comparisons suggest that each grain prefers one phenolic acid synthesis pathway to the others, leading to a unique phenolic acid profile. Quinoa appears to have significantly fewer phenolic acids than other grains, most likely as a result of their low synthesis. To validate these findings, further research and discussion are needed.

#### 3.2.2. Health Potential

Epidemiological evidence suggests that phenolic acids have remarkable health-promoting impacts on chronic diseases, such as anti-inflammatory, antioxidant, antidiabetic, anticarcinogenic, and others (Table 6). One important function of phenolic acids is their ability to prevent cancer by preventing normal cell transformation, tumor growth, angiogenesis, and metastasis, all of which are factors that contribute to the development and spread of cancer. Moreover, phenolic acids promote the production of proteins that limit tumor growth, including phosphatase, p53, and the tensin homolog PTEN [107]. Several investigations have demonstrated that *p*-coumaric acids have antibacterial, anti-inflammatory, and anticancer properties [108,109,110]. For instance, Janicke et al. [110] found that *p*-coumaric acid inhibited the growth of Caco-2 colon cancer cells in the cell cycle, hence protecting against the development of colon cancer. Feruloyl-L-arabinose reduced lung cancer cell penetration, motility, and the formation of reactive oxygen species. Other phenolic acids, such as ferulic, feruloyl-L-arabinose, and *p*-coumaric, have also been examined in various cell lines for their anti-inflammatory, anticarcinogenic, antihypertensive, and antidiabetic potential [111,112,113,114]. According to Fahrioğlu et al. [112], ferulic acid displayed anticancer properties via influencing the cell cycle, invasion, and apoptotic behavior of MIA PaCa-2 cells. In addition, Eitsuka et al. [115] investigated the anticancer properties of ferulic acid against cancer cell proliferation; the authors found that the combination of the two compounds inhibited the proliferation of MCF-7, PANC-1, and DU-145 cells more effectively than individual ones alone. Moreover, numerous studies have shown that consuming whole grains rich in phenolic acids protects against a number of cardiovascular and blood-circulation-related diseases, including caffeic acid, while also improving insulin resistance, plasma triglyceride levels, and platelet function, demonstrating anticarcinogenic and antimutagenic properties [116].

Furthermore, no comprehensive investigation on the biological activity of sinapic acid can be found. A literature search revealed the existence of both free and ester forms of sinapic acid, with some examples of esters being sinapoyl esters, sinapine (sinapoylcholine), and sinapoyl malate [117]. Spices, citrus and berry fruits, vegetables, cereals, oilseed crops, and vegetables are among the edible plants that contain the phytochemical sinapic acid [118,119]. Sinapic acid has been investigated and documented in relation to a number of clinical conditions, including infections, oxidative stress [120], inflammation [121], cancer [122], T2D [123], neurodegeneration [124], and anxiety [125]. Studies have also been conducted on the acetylcholinesterase inhibition [126,127], antimutagenic [128], and antioxidant activity [129] of a few sinapic acid derivatives, including sinapine, 4-vinylsyringol, and syringaldehyde. In regard to *p*-hydroxybenzoic acid, we examined the antithrombogenic, anticoagulation, and inhibitory effects of protocatechuic acid, isovanillic acid, and 4-hydroxybenzoic acid, which all function as antithrombotic and anticoagulant agents [130,131]. However, there are no studies on how these substances affect blood cell viability or their inhibitory effects on fibrin clot formation, plasma recalcification, or the enzymatic activity of procoagulant proteases or fibrinoligases. DU-145 human prostate carcinoma cells and human leukemia (HL)-60 cancer cells are two examples of cancer cell types on which gallic acid has been shown to have a strong anticancer impact in a number of studies [132,133]. Furthermore, human epidermoid carcinoma (A431) skin cancer cells are not able to proliferate when methyl gallate is present [112,134]. In addition, it has been demonstrated that phenolic acids can be considered excellent antioxidants that are capable of neutralizing excessive damage to the body produced by free radicals and chronic conditions. The antioxidant capacity of hydroxybenzoic acids is centered on phenolic hydroxyl. In addition, methoxy and carboxy groups have a significant impact on phenolic acids’ antioxidant capabilities. They have an important role in the prevention of Alzheimer’s and Parkinson’s diseases, both of which are neurological illnesses, as well as vascular dementia and cerebrovascular insufficiency syndromes [135,136,137]. They also have antidiabetic, anticancer, cardioprotective, and anti-inflammatory properties [138]; see Table 5.

**Table 6:** Hydroxycinnamic acids and hydroxybenzoic acids and their health potential.

Phenolic Acid	Health Potential	References
*p*-Coumaric acid	Antimicrobial, Anti-inflammatory, anticancer	[108,109,110]
Ferulic acid	Anticancer, antihypertensive, antidiabetes, anti-inflammatory	[111,112,113,114,115]
Caffeic acid	Antimutagenic, anticarcinogenic	[116]
Sinapic acid	Antioxidative, anti-inflammatory, anticancer, antidiabetic, anti-neurodegeneration, anti-anxiety	[120,121,122,123,124,125]
*p*-Hydroxybenzoic acid	Antithrombotic and anticoagulant	[130,131]
Gallic acid	Anticancer, HCV inhibition, antibacterial	[132,133,134]
Vanillic acid	Alzheimer’s disease and Parkinson’s disease, neurological disorders, vascular dementia, anti-inflammatory, and cerebrovascular insufficiency states	[135,136,137]
Syringic acid	Antidiabetic, anticancer, cardioprotective, anti-inflammatory	[138]

Table 6 summarizes the phenolic acids found in grains and their health potential. With regard to *p*-hydroxybenzoic acid intake, several epidemiological studies support its protective effects against bacterial disorders, cardiovascular disease, type 2 diabetes, neurodegenerative diseases, and cancer. Numerous studies have been performed to examine the anti-platelet aggregation and antithrombotic activity of isovanillic acid and *p*-hydroxybenzoic acid, which act as antithrombotic and anticoagulant agents; however, no research has been conducted on how these compounds affect the viability of blood cells or how they inhibit the development of fibrin clots, the enzymatic activity of procoagulant proteases or fibrinoligases, and plasma recalcification. Furthermore, there is no extensive research on the biological properties of sinapic acid in the literature. Moreover, there is a lack of comprehensive literature regarding the biological characteristics of sinapic acid. This short review article summarizes these findings so that the scientific community may focus more on the biological properties of sinapic acid. Furthermore, cereal grains’ phenolic compounds have been shown to inhibit Parkinson’s and Alzheimer’s disease, as well as having anti-analgesic, anti-allergic, cardioprotective, and antidiabetic properties. As a result, phenolic compounds are considered to be beneficial natural bioactive and nutraceutical agents that can be used to prevent or inhibit a number of chronic non-communicable conditions, including inflammation.

## 4. Effect of Consuming Whole Grains on Inflammatory Active Components

### 4.1. Evidence from Epidemiological Studies

Epidemiological data strongly support the findings that the consumption of whole grains may reduce the risk of inflammation [4,5,6,7,15,21,22,23], as well as being beneficial in several other diseases, like coronary heart disease, CVD, T2D, and cancer [18,19,20,63,139]. Several studies support the role of whole grain dietary fiber and phenolic compounds in inflammation [81,83,111,121,138]. Research suggests multiple mechanisms of action, which remain unclear. The significance of subclinical inflammation is increasingly attracting attention in the literature as a common denominator in most disease processes [140,141]. 

In this regard, Xu et al. [142] collected data from different randomized controlled trial studies to investigate the relationship between whole grain intake and circulating inflammation markers. This meta-analysis included data on 838 individuals from nine RCTs. The findings of these analyses revealed that alterations in the inflammatory markers CRP, IL-6, TNF-α, and IL-1β were negatively correlated with higher whole grain intake intake (standardized mean difference, 0.16; 95% confidence interval, 0.02–0.30. Furthermore, whole grain consumption was found to be inversely associated with a significant decrease in the levels of IL-6 (standardized mean difference, 0.19; 95%CI, 0.03–0.36) and CRP (standardized mean difference, 0.29; 95%CI, 0.08–0.50). Moreover, a substantial reduction in the levels of CRP and IL-6 was observed to be negatively correlated with whole grain intake. A meta-analysis of 13 trials and 466 individuals revealed that the consumption of whole grains led to a significant increase in blood concentrations of hs-CRP and IL-6, but had no significant effect on TNF-α levels [143].

Recently, Zamaratskaia et al. [144] conducted an intervention trial study on the effect of a whole grain diet on low-grade inflammatory biomarkers in men with prostate cancer. This study examined the effects of whole grain/bran rye consumption on low-grade inflammation and endothelial function biomarkers in men with prostate cancer. In a randomized crossover design, seventeen men with untreated, low-grade prostate cancer consumed 485 g of whole grain and bran products or refined wheat products with added cellulose daily. Fasting blood samples were collected before and after two, four, and six weeks of treatment. In comparison to diets containing refined wheat products, males with prostate cancer who consumed whole grains including rye and rye bran had lower levels of the inflammatory biomarkers TNF receptor 2, E-selectin, and endostatin. A beneficial impact of whole grain intake and subclinical inflammation was reported by [145], when 50 obese participants (body mass index > 30 kg/m^2^) consumed whole-grain-rich meals for a period of 12 weeks. The authors discovered that the CRP concentrations in the whole grain group had significantly decreased, by 38%, at the end of the intervention, while the concentrations in the refined grain group remained unchanged [145].

Summarizing these benefits, numerous systematic reviews and meta-analyses have been published that validate these findings and are now collected into “umbrella reviews” [146,147,148]. Whole grains have been shown in studies to enhance glucose kinetics, reduce peripheral insulin resistance, and lower inflammation [6,7,142,149]. However, it should be noted that whole grain consumption may not be the only factor contributing to health in general; other factors include smoking less, drinking less alcohol, and being more physically active [150,151].

### 4.2. Dietary Fiber

**Figure 1:**
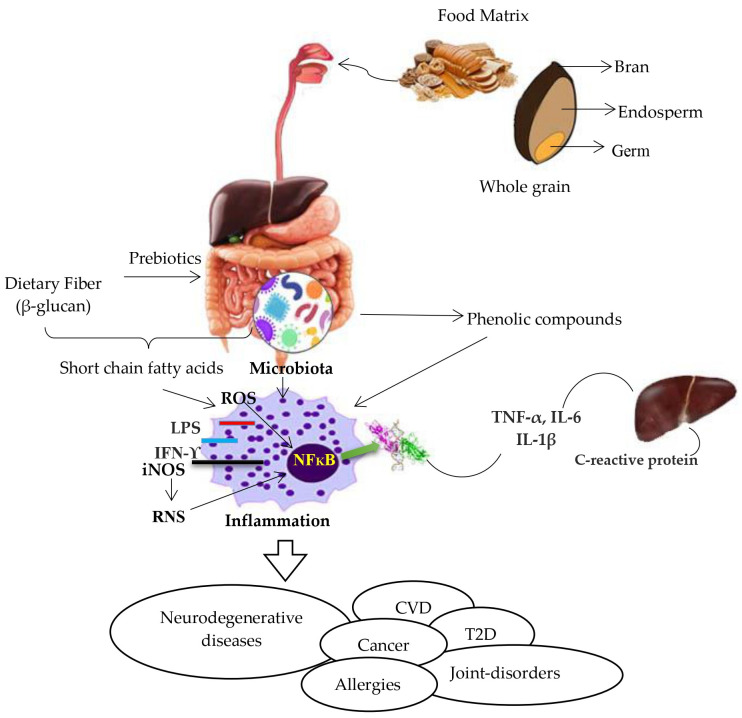
Connections between whole grain dietary fiber and phenolic compounds and inflammation; role of gut microbiota, interferon gamma (IFN-ϒ), interleukin 1 beta (IL-1β), interleukin 6 (IL-6), inducible nitric oxide synthase (iNOS), lipopolysaccharide (LPS), nuclear factor kappa-light-chain-enhancer of activated B cells (NFκB), reactive nitrogen species (RNS), reactive oxygen species (ROS), and tumor necrosis factor alpha (TNF-α).

Whole grains are high in SDF, particularly β-glucan, which is considered to be a major active component of whole grains that alters and stimulates the gut bacteria (Figure 1). Increased dietary fiber consumption has been shown to provide a number of health benefits, including anti-inflammatory properties. Additional evidence suggests a direct link between carbohydrate-rich and dietary-fiber-deficient diets in the development of inflammation, lending support to the important role of dietary fiber in inflammation [152]. Furthermore, Qi et al. [153] investigated the relationship between whole grain dietary fiber and inflammatory markers and found an inverse correlation between the highest 18% and lowest 8% quintiles of cereal fiber consumption and lower levels of CRP and TNF receptor 2. Whole grain products with β-glucan have also been investigated for their impacts on ulcerative colitis [81], a severe inflammatory bowel disease characterized by gastrointestinal inflammation and induced by persistent or recurrent immune system activation, and the upregulation of pro-inflammation markers, such IL-1β, has been observed in animal models [82]. Most research on colitis focuses on the pro-inflammatory biomarker IL-1β, which is largely generated by lamina propria monocytes and macrophages that infiltrate the colitis mucosa [83]. In another comparable study, to evaluate their anti-inflammatory properties, male Wistar rats were fed diets containing two whole grain barley cultivars with varied dietary fiber and β-glucan content; it also studied the solubility of dietary fiber [76]. The authors discovered that the consumption of barley may reduce the risk of inflammation, as shown by lower plasma levels of LPS-binding protein and monocyte chemoattractant protein 1.

In a study involving 80 mice, Liu et al. [77] found that oat β-glucan might prevent colitis caused by dextran sodium sulfate. This study found that the consumption of oat β-glucan at 500 and 1000 mg/kg lowered the aberrant mRNA expression of inflammatory biomarkers, including TNF-α, IL-6, and IL-1β. In contrast, Wilczak et al. [154] evaluated the influence of two forms of β-glucan, high and low, on 72 male Sprague-Dawley rats with LPS-induced enteritis. This study discovered that β-glucan, especially low-molecular-weight oat β-glucan, has potent anti-inflammatory properties, as demonstrated by anti-inflammatory markers, pro-inflammatory IL-12, TNF-α, and the profiles of both the lamina propria and intraepithelial lymphocyte populations that inhabit the colon tissue.

As previously mentioned, dietary fiber is likely to affect the diversity of the colonic microbiota and the production of short-chain fatty acids (SCFAs). Additionally, butyrate is known to mediate inflammatory pathways; dietary fiber has an impact on the inflammatory status both systemically and inside the colon, since butyrate is known to mediate the inflammatory pathways. Subsequent investigations should focus on the involved mechanisms. It will be challenging to determine the precise mechanisms that mediate dietary fiber’s anti-inflammatory effects, since dietary fiber has many potential benefits, several of which are discussed in this section, and because the mechanisms underlying these benefits are complex and may involve the colonic microflora. Thus, numerous strategies, such as prospectively designed randomized controlled trials and rodent-based mechanistic investigations, will be needed for future study.

### 4.3. Phenolic Compounds

Inflammation is an important biological reaction to any tissue damage. Pro-inflammatory cytokines are released by the immune system in response to any stimulation, including injury, infection, or irritation [155]. Pro-inflammatory cytokine overproduction, such as that of TNF-α and IL-1b, can cause major health conditions, such as allergies, cancer, joint problems, and cardiovascular disease. Therefore, it is essential to reduce the overproduction of these pro-inflammatory cytokines in order to prevent and control these diseases. Since ancient times, phenolic chemicals have been used to treat inflammation and associated diseases. To further understand the influence of whole grains on inflammation, numerous bioactive components of whole grains and their metabolites have been studied for their potential effects on inflammation indicators [17,156]. Although prior research has mostly focused on the presence of fiber in whole grains for its anti-inflammatory characteristics, recent findings indicate that the anti-inflammatory substances in whole grains extend beyond fiber [157,158]. Other bioactive compounds found in whole grains have also been linked to their anti-inflammatory properties. In this regard, [159] studied the anti-inflammatory activity of *p*-coumaric acid by assessing the TNF-α levels in arthritic rats’ synovial tissue. *p*-Coumaric acid possessed anti-inflammatory properties and decreased TNF-α expression. In addition, [111] reported that ellagic and caffeic acids have anti-inflammatory properties. Mice were fed both caffeic acid and ellagic acid at the ratio of 2.5 and 5.0% by mixing them into their usual food. The expression of inflammatory mediators was decreased upon the inclusion of these phenolic acids. In a high-fructose-diet-mediated metabolic alteration model, caffeic acid reduced pro-inflammatory cytokines such as serum IL-6, IL-8, and TNF-α, resulting in anti-inflammatory, anti-hyperglycemic, and anti-hyperlipidemia effects [18]. Interestingly, Ibitoye et al. [160] discovered that, via increasing NO bioavailability, caffeic acid positively modulated blood pressure in rats with cyclosporine-induced hypertension.

Whole grains contain avenantramides (AVAs), which are secondary metabolites. These polyphenolic compounds, which are found in the bran portion of oat grains and are crucial components of oat groats, have also been investigated in relation to inflammation. The three most prevalent AVAs in oats are esters of 5-hydroxyanthranilic acid with *p*-coumaric acid, ferulic acid, or caffeic acid. Oats’ phenolic compounds, including AVAs, have also been shown to have anti-inflammatory and antioxidant properties. It has been shown in both in vivo and in vitro investigations to significantly decrease the inflammatory response in the systemic circulation caused by exercise in women of all ages [161]. This study found that treatment with AVA reduced the levels of NFκB activation, CRP, IL-1β, IL-6, and neutrophil respiratory bursts, using a human aortic endothelium cell culture system. Liu et al. [162] investigated the anti-inflammatory and anti-atherogenic characteristics of oat AVAs. After subjecting human aortic endothelial cells to AVA pretreatment for a whole day, the amount of IL-1β-induced inflammation was notably decreased. Furthermore, Sur et al. [163] investigated the AVAs’ anti-inflammatory properties in human keratinocytes. According to the study findings, NF-κB-dependent luciferase activity was dramatically reduced by AVA treatment, which also resulted in a decrease in α-IL-8 release in keratinocytes treated with TNF-α.

Ex vivo studies have demonstrated the anti-inflammatory properties of phenolic acids and their metabolites in the circulation. Mateo Anson et al.’s study [164] examined the impact of phenolic acids and their metabolites on biomarkers associated with inflammation in eight healthy males. They were given a low-phenolic-acid diet for three days, followed by an overnight fast, and then they were given 300 g of either low-phenolic-acid regular wheat bread or high-phenolic-acid whole wheat bioprocessed bread. Following bread consumption, at 0, 1.25, 6, and 12 h, blood samples were taken and subjected to LPS incubation. After consuming bioprocessed wheat bread with a high phenolic acid concentration versus bread with low phenolic acid content, a comparison of several blood samples showed a considerably reduced pro-inflammatory to anti-inflammatory cytokine ratio.

In summary, studies on whole grains’ impacts on inflammation have shown conflicting results, most likely as a result of the various study methodologies and designs. For instance, several trials were less controlled than others, despite the fact that they maintained the participants’ weight, strictly monitored the food, and kept the examined dietary components consistent. Furthermore, the apparent impact of whole grains on inflammatory markers may have been influenced by the exclusion of biomarkers of adherence from some study designs. Additionally, although some research was performed on healthy people who were not likely to have a weak immune system or a high inflammatory status, it is possible that the participants who were preselected due to having a chronic disease or a high inflammatory status showed more noticeable changes. Therefore, in order to sufficiently prove the therapeutic efficacy of phenolic acids and establish their safety for human intake, more research and clinical trials are necessary.

### 4.4. Proposed Mechanism of Anti-Inflammatory Properties of Whole Grain Dietary Fiber and Phenolic Compounds and Involvement of Gut Microbiota

Whole grains’ potential to modulate inflammation has been attributed to SCFAs, which are byproducts of the microbial degradation of whole grain dietary fiber (Figure 1). The anti-inflammatory properties of whole grains may be partially attributed to the bioactive properties of SCFAs [77,153,154]. Whole grain consumption has been shown to enhance the concentration of *Lachnospira*, a producer of SCFAs, as well as the stool SCFA content, when compared to refined grain consumption [165]. SCFAs (butyrate, propionate, and acetate) in the blood and gastrointestinal tract can influence leukocyte activity and cytokine synthesis, which can reduce inflammation. Acting via signaling pathways like NF-κB and mitogen-activated protein kinase, cytokines are soluble regulatory signals and intercellular messengers that both trigger and limit inflammatory responses. When cytokines reach areas of inflammation, they eliminate microbial pathogens [166]. Although cytokines play a vital role in directing both the innate and adaptive immune responses to inflammation, an excessive amount can be harmful. Furthermore, the activity of histone deacetylase may be impacted by SCFAs. It has been demonstrated that histone deacetylase inhibitors decrease ex vivo reactions to pro-inflammatory stimuli, as well as the plasma levels of cytokines [167,168]. According to a number of recently published studies, the capacity of SCFAs to stimulate immune cells aids in the elimination of pathogens that cause inflammation and regulates the overproduction of cytokines and chemokines.

Numerous investigations have demonstrated the anti-inflammatory and antioxidant qualities of phenolic compounds [97,102,111,120,121,129,159]. Supplementation with phenolic acids has been shown significantly reduce the pro-inflammatory response to exercise-induced inflammation [161]. The authors revealed that treatment with phenolic acids reduced neutrophil respiratory bursts, CRP, IL-1β, and IL-6 levels, and NFκB activation; see Figure 1. Furthermore, Liu et al. [162] studied the impact of whole grain phenolic acids in a human aortic endothelial cell culture in terms of anti-inflammatory and anti-atherogenic properties. According to this study, endothelial cells treated with phenolic acids for 24 h showed significantly reduced IL-1β-induced inflammation. Additionally, Sur et al. [163] investigated the potential anti-inflammatory properties of phenolic acids in human keratinocytes. The results of this investigation showed that the phenolic acids significantly inhibited NF-κB-dependent luciferase activity and reduced IL-8 release in TNF-α-treated keratinocytes.

Whole grains may have some beneficial effects on the gut microbiome due to their ability to influence both the host metabolism and gut microbial ecology (Figure 1). Consequently, a number of nutritional studies have examined the effect of whole grains on inflammation, including the role of the gut microbiome. In addition, according to Lee et al. [169], whole grains have modulatory effects on inflammation markers; they also studied the changes in the populations of useful microbiota such as *Lactobacillus* and *Bifidobacterium*, as well as observing a lower abundance of the *Bacteroides fragilis* bacterial group in the cecum. Moreover, Vitaglione et al. [35] discovered a link between a significant decrease in the inflammatory marker TNF-α and an increase in *Lactobacillus* and *Bacteroides* spp. abundance. Furthermore, Martínez et al. [170] examined whether the gut microbiota was connected to the effect of whole grains on inflammatory markers. This study included 28 healthy adult mice that consumed 60 g of whole grain barley, brown rice, or a combination of the two, every day for four weeks. The findings of this investigation demonstrated a reduction in plasma IL-6 levels as well as a potential decrease in plasma hs-CRP levels. High-fat diet supplementation had the opposite effect on the alterations generated by the high-fat diet in mice and had a “prebiotic effect,” comparable to improving the number of *Lactobacillus*, *Bifidobacterium*, *Akkermansia*, and *Bacteroides-Prevotella*. Furthermore, combining whole grains and bran with a high-fat diet improved the amount of *Roseburia* spp., an essential butyrate-producing bacterium in the gut that may promote butyrate-dependent anti-inflammatory effects. Meanwhile, a high-fat diet reduced the levels of Enterobacteriaceae, which may have been connected to a reduction in LPS translocation and an improvement in gut barrier integrity, and such prebiotic actions may play a role in the anti-inflammatory and metabolic benefits. Thus, whole grains appear to regulate inflammation through different pathways. These beneficial actions might include the positive effects of dietary fiber, phenolic acids and their metabolites, and the microbial metabolites SCFAs (related to fiber). While some of these advantages are direct, others are due to prebiotic effects on gut bacteria, which convert important bioactive substances into more beneficial metabolites, influencing the biomarkers associated with inflammation.

## 5. Conclusions

This manuscript presents a comprehensive review of the composition and biological activity of whole grain dietary fiber and phenolic acids. As reviewed above, whole grains contain various types of dietary fiber and phenolic acids; however, different grains typically have a distinct dietary fiber and phenolic acid profile. The benefits of dietary fiber and phenolic acids from whole grains have been extensively studied throughout the years. The bran and germ, which are removed during the grain refinement process, contain the majority of the components that are beneficial to health. Emerging data suggest that whole grain consumption has benefits beyond basic nutrition, which is supported by epidemiological research revealing a protective impact of whole grains against inflammation. The anti-inflammatory activity may involve the impact of dietary fiber, microbial metabolites such as SCFAs, and phenolic acids. While some of these benefits are direct, others involve the prebiotic’s action on the gut bacteria, which convert essential bioactive compounds into more beneficial metabolites, hence influencing inflammatory biomarkers. Challenges appear in diverse areas; therefore, it is critical to determine which of these components may have the greatest protective action. Further research is needed to understand their effects fully, whether the bran, germ, dietary fiber, or specific phenolic acids associated with reducing the levels of inflammatory markers.

In general, whole grains contain both dietary fiber and phenolic acids. The modulatory effects of whole grains on inflammation might be exerted via several mechanisms, including the effects of dietary fiber and phenolic compounds and the role of the gut microbiota. While beyond the scope of the study, it is worth mentioning that the intestinal mycobiome and virome are also important. Candida of the gut has been studied in relation to many diseases and also with respect to dietary contributions. However, more direct evidence linking these mechanisms to the observed health outcomes would strengthen the argument.

## Figures and Tables

**Table 1:** Targeted cereals used for the study.

**Cereal**	**Botanical Name**
Rye	*Secalecereale* L.
Corn	*Zea mays* L.
Sorghum	*Sorghum bicolor*
Millets	*Eleusine coracana* (L.) Gaertn.
Triticale	*Triticosecale* Wittmack
**Pseudo-Cereal**	**Botanical Name**
Quinoa	*Chenopodium quinoa* Willd.

**Table 3:** Dietary fiber in whole grains and their health potential.

Grain	Dietary Fiber	Health Potential	References
Whole grains	Arabinoxylans	Enhance fecal biomass, improve gut health, lower LDL levels, lipid metabolism	[71,72,73,74,75]
Whole grains	β-glucan	Anti-inflammation, decrease glycemic index, prebiotic effect, decrease blood lipids, modulate blood cholesterol and glucose levels, immune function	[69,70,76,77,78,79]
Whole grains	Total dietary fiber	Anti-inflammation, anti-cardiovascular disease, antidiabetic, certain anticancer effects, body weight regulation	[67,80,81,82,83]

**Table 4:** Chemical structures of targeted hydroxycinnamic acids and hydroxybenzoic acids.

**Hydroxycinnamic Acids**	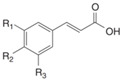			
	**R_1_**	**R_2_**	**R_3_**	**Chemical Structure**
*p*-Coumaric acid	H	OH	H	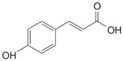
Ferulic acid	H	OH	OCH_3_	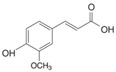
Caffeic acid	OH	OH	OH	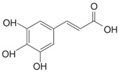
Sinapic acid	OCH_3_	OH	OCH_3_	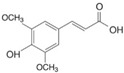
**Hydroxybenzoic Acids**	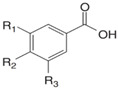			
	**R_1_**	**R_2_**	**R_3_**	
*p*-Hydroxybenzoic acid	H	OH	H	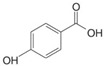
Gallic acid	OH	OH	OH	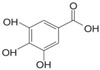
Vanillic acid	H	OH	OCH_3_	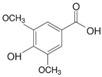
Syringic acid	OCH_3_	OH	OCH_3_	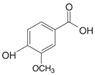

**Table 5:** Review of hydroxycinnamic acids and hydroxybenzoic acids in targeted cereals, µg/g of dry weight.

**Hydroxycinnamic Acids**					
**Whole Grain**	**Ferulic Acid**	***p*-Coumaric Acid**	**Caffeic Acid**	**Sinapic Acid**	**References**
Rye (*Secalecereale* L.)	827.2 (218.7–1170.0)	49.0 (29.9–70.0)	16.2 (12.3–20.0)	94.2 (51.7–140.0)	[91,102]
Corn (*Zea mays* L.)	94.2 (51.7–140.0)	340.5 (97.0–584.0)	15.0 (5.7–24.4)	66.1 (52.9–79.3)	[89,91,94,100,101]
Sorghum (*Sorghum bicolor*)	66.1 (52.9–79.3)	43.6 (3.8–83.4)	32.1 (1.9–62.4)	8.2	[95]
Millet (*Eleusine coracana* (L.) Gaertn.)	233.4 (20.0–571.3)	46.0 (18.0–118.3)	4.6 (1.1–8.2)	46.7 (21.3–72.1)	[96,97,98]
Triticale (*Triticosecale* Wittmack)	46.7 (21.3–72.1)	139.8 (21.2–258.5)	9.9 (6.0–13.9)	83.0 (50.0–140.0)	[91,101]
Quinoa (*Chenopodium quinoa* Willd.)	87.7 (23.7–150.0)	48.6 (17.1–80.0)	7.0	-	[99,100]
**Hydroxybenzoic Acids**					
	***p*-Hydroxybenzoic Acid**	**Gallic Acid**	**Vanillic Acid**	**Syringic Acid**	
Rye (*Secalecereale* L.)	14.1 (8.1–20.0)	7.7	18.0 (15.9–20.0)	6.3	[91]
Corn (*Zea mays* L.)	8.2 (4.9–11.6)	55.4 (0.5–116.5)	10.3 (5.4–15.4)	45.3 (4.3–108.4)	[91,101]
Sorghum (*Sorghum bicolor*)	36.2 (6.1–148.0)	28.0 (13.2–46.0)	23.2 (8.3–50.7)	16.9 (15.6–19.7)	[94,104]
Millet (*Eleusine coracana* (L.) Gaertn.)	3.0	68.6 (38.7–109.0)	22.2 (11.0–33.3)	13.1 (2.1–24.0)	[89,96,97]
Triticale (*Triticosecale* Wittmack)	7.1 (6.9–7.4)	333.7 (123.4–544.0)	446.0 (154.0–738.0)	173.2 (5.3–341.0)	[91,101]
Quinoa (*Chenopodium quinoa* Willd.)	21.7 (13.8–29.0)	-	30.4 (11.7–110.0)	-	[100]

## Data Availability

All the data are already provided in the main manuscript.
